# 2-Azido-1-(4-fluoro­phen­yl)ethanone

**DOI:** 10.1107/S1600536812013426

**Published:** 2012-03-31

**Authors:** Sammer Yousuf, Muhammad Arshad, Hafiza Madiha Butt, Sumayya Saeed, Fatima Z. Basha

**Affiliations:** a H.E.J. Research Institute of Chemistry, International Center for Chemical and Biological Sciences, University of Karachi 75270, Pakistan; b Department of Chemistry, University of Karachi 75270, Pakistan

## Abstract

The crystal structure of the title compound, C_8_H_6_FN_3_O, is stabilized by C—H⋯O hydrogen bonds, which link the mol­ecules into chains running parallel to the *a* axis.

## Related literature

The title compound is an inter­mediate obtained during an attempt to synthesize biologically active triazoles. For the biological activity of triazoles, see: Genin *et al.* (2000 ▶); Parmee *et al.* (2000 ▶); Koble *et al.* (1995 ▶); Moltzen *et al.* (1994 ▶). For standard bond lengths: Allen *et al.* (1987 ▶).
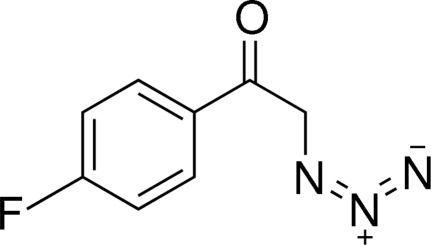


## Experimental

### 

#### Crystal data

C_8_H_6_FN_3_O
*M* *_r_* = 179.16
Orthorhombic, 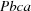

*a* = 10.7985 (16) Å
*b* = 8.3971 (12) Å
*c* = 17.485 (3) Å
*V* = 1585.5 (4) Å^3^
*Z* = 8
Mo *K*α radiation
μ = 0.12 mm^−1^
*T* = 273 K
0.35 × 0.28 × 0.20 mm

#### Data collection

Bruker SMART APEX CCD area-detector diffractometer
Absorption correction: multi-scan (*SADABS*; Bruker, 2000 ▶) *T*_min_ = 0.959, *T*_max_ = 0.976
8606 measured reflections
1476 independent reflections
1239 reflections with *I* > 2σ(*I*)
*R*_int_ = 0.034

#### Refinement

*R*[*F* ^2^ > 2σ(*F* ^2^)] = 0.033
*wR*(*F* ^2^) = 0.090
*S* = 1.06
1476 reflections
118 parameters
H-atom parameters constrained
Δρ_max_ = 0.20 e Å^−3^
Δρ_min_ = −0.19 e Å^−3^

### 

Data collection: *SMART* (Bruker, 2000 ▶); cell refinement: *SAINT* (Bruker, 2000 ▶); data reduction: *SAINT*; program(s) used to solve structure: *SHELXS97* (Sheldrick, 2008 ▶); program(s) used to refine structure: *SHELXL97* (Sheldrick, 2008 ▶); molecular graphics: *SHELXTL* (Sheldrick, 2008 ▶); software used to prepare material for publication: *SHELXTL* *PARST* (Nardelli, 1995 ▶) and *PLATON* (Spek, 2009 ▶).

## Supplementary Material

Crystal structure: contains datablock(s) global, I. DOI: 10.1107/S1600536812013426/rz2730sup1.cif

Structure factors: contains datablock(s) I. DOI: 10.1107/S1600536812013426/rz2730Isup2.hkl

Supplementary material file. DOI: 10.1107/S1600536812013426/rz2730Isup3.cml

Additional supplementary materials:  crystallographic information; 3D view; checkCIF report

## Figures and Tables

**Table 1:** Hydrogen-bond geometry (Å, °)

*D*—H⋯*A*	*D*—H	H⋯*A*	*D*⋯*A*	*D*—H⋯*A*
C1—H1*B*⋯O1^i^	0.93	2.45	3.3722 (17)	174

Symmetry code: (i) 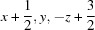.

**Fig. 1.**
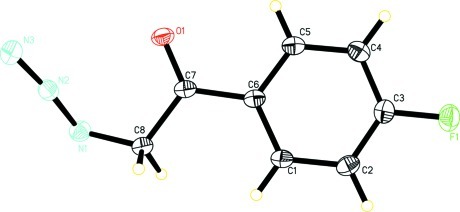
The molecular structure of the title compound with displacement ellipsoids drawn at 30% probability level.

**Fig. 2.**
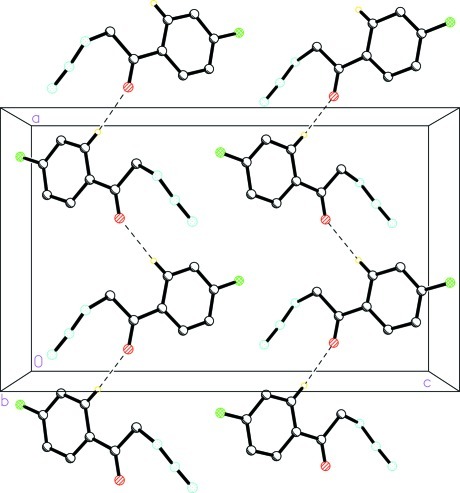
Crystal packing of the title compound viewed along the b axis. Hydrogen atoms not involved in hydrogen bonds (dashed lines) are omitted.

## supplementary crystallographic information

### Comment

The synthesis of 1,2,3-trizoles *via* click chemistry approach has gained much attention by synthetic chemists due to their immensely known medicinal importance (Genin *et al.*, 2000, Parmee *et al.*, 2000, Koble *et al.*, 1995, Moltzen *et al.*, 1994). In our quest for the synthesis of therapeutically active triazoles starting from commercially available acetophenone derivatives, a number of azides including the title compound, whose crystal structure is reported herein, have been prepared as an intermediate.

In the molecule of the title compound (Fig. 1), the bond lengths (Allen *et al.*, 1987) and angles are within normal ranges. The crystal structure (Fig. 2) is stabilized by intermolecular hydrogen bonds (Table 1) linking the m olecules to form chains parallel to the *a* axis.

### Experimental

1-(4-Fluorophenyl)ethanone (7.239 mmol, 1.0 equiv.) was dissolved in acetonitrile (18 ml) in a round bottom flask. To the stirred mixture, *p*-toluenesulphonic acid (10.858 mmol, 1.5 equiv.) and *N*-bromosuccinimide (10.134 mmol, 1.4 equiv.) were added, and the solution was heated to reflux for 1 to 1.5 h until completion of the reaction as monitored by TLC analysis. The reaction mixture was cooled to room temperature and sodium azide (21.717 mmol, 3.0 equiv.) was added. After additional stirring for 2 to 3 h, ice cooled water was added to quench the reaction. The reaction mixture was extracted with diethyl ether (2 × 25 ml) and the combined organic layer was dried over anhydrous Na_2_SO_4_, filtered and concentrated in vacuum to get the crude product. The crude product was purified by flash silica gel chromatography (EtOAc/hexane, 1:9–3:7 *v*/*v*) to afford the title compound in 65% yield. Recrystallization by slow evaporation of an ethanol solution afforded crystals suitable for single-crystal X-ray studies. All chemicals were purchased from Sigma-Aldrich.

### Refinement

H atoms were positioned geometrically with C—H = 0.93–0.97 Å, and constrained to ride on their parent atoms with *U*_iso_(H) = 1.2 *U*_eq_(C).

### Crystal data

**Table:**

C_8_H_6_FN_3_O	*F*(000) = 736
*M**_r_* = 179.16	*D*_x_ = 1.501 Mg m^−^^3^
Orthorhombic, *P**b**c**a*	Mo *K*α radiation, λ = 0.71073 Å
Hall symbol: -P 2ac 2ab	Cell parameters from 2831 reflections
*a* = 10.7985 (16) Å	θ = 2.3–27.8°
*b* = 8.3971 (12) Å	µ = 0.12 mm^−^^1^
*c* = 17.485 (3) Å	*T* = 273 K
*V* = 1585.5 (4) Å^3^	Block, colourless
*Z* = 8	0.35 × 0.28 × 0.20 mm

### Data collection

**Table:**

Bruker SMART APEX CCD area-detector diffractometer	1476 independent reflections
Radiation source: fine-focus sealed tube	1239 reflections with *I* > 2σ(*I*)
Graphite monochromator	*R*_int_ = 0.034
ω scan	θ_max_ = 25.5°, θ_min_ = 2.3°
Absorption correction: multi-scan (*SADABS*; Bruker, 2000)	*h* = −10→13
*T*_min_ = 0.959, *T*_max_ = 0.976	*k* = −10→10
8606 measured reflections	*l* = −21→21

### Refinement

**Table:**

Refinement on *F*^2^	Primary atom site location: structure-invariant direct methods
Least-squares matrix: full	Secondary atom site location: difference Fourier map
*R*[*F*^2^ > 2σ(*F*^2^)] = 0.033	Hydrogen site location: inferred from neighbouring sites
*wR*(*F*^2^) = 0.090	H-atom parameters constrained
*S* = 1.06	*w* = 1/[σ^2^(*F*_o_^2^) + (0.0469*P*)^2^ + 0.2956*P*] where *P* = (*F*_o_^2^ + 2*F*_c_^2^)/3
1476 reflections	(Δ/σ)_max_ < 0.001
118 parameters	Δρ_max_ = 0.20 e Å^−^^3^
0 restraints	Δρ_min_ = −0.19 e Å^−^^3^

### Special details

**Table:**

Geometry. All e.s.d.'s (except the e.s.d. in the dihedral angle between two l.s. planes) are estimated using the full covariance matrix. The cell e.s.d.'s are taken into account individually in the estimation of e.s.d.'s in distances, angles and torsion angles; correlations between e.s.d.'s in cell parameters are only used when they are defined by crystal symmetry. An approximate (isotropic) treatment of cell e.s.d.'s is used for estimating e.s.d.'s involving l.s. planes.
Refinement. Refinement of *F*^2^ against ALL reflections. The weighted *R*-factor *wR* and goodness of fit *S* are based on *F*^2^, conventional *R*-factors *R* are based on *F*, with *F* set to zero for negative *F*^2^. The threshold expression of *F*^2^ > σ(*F*^2^) is used only for calculating *R*-factors(gt) *etc*. and is not relevant to the choice of reflections for refinement. *R*-factors based on *F*^2^ are statistically about twice as large as those based on *F*, and *R*- factors based on ALL data will be even larger.

### Fractional atomic coordinates and isotropic or equivalent isotropic displacement parameters (Å^2^)

**Table:**

	*x*	*y*	*z*	*U*_iso_*/*U*_eq_	
O1	0.61761 (8)	0.03188 (12)	0.73973 (5)	0.0358 (3)	
N1	0.77319 (12)	−0.13811 (15)	0.83252 (7)	0.0361 (3)	
N2	0.68380 (11)	−0.09577 (14)	0.87237 (7)	0.0336 (3)	
N3	0.60363 (13)	−0.07646 (17)	0.91283 (7)	0.0451 (4)	
C1	0.89336 (12)	0.19093 (16)	0.64990 (8)	0.0305 (3)	
H1B	0.9536	0.1521	0.6830	0.037*	
C2	0.92779 (14)	0.28254 (17)	0.58742 (8)	0.0348 (4)	
H2A	1.0106	0.3060	0.5782	0.042*	
C3	0.83684 (14)	0.33759 (17)	0.53966 (8)	0.0342 (3)	
C4	0.71318 (14)	0.30861 (18)	0.55138 (8)	0.0366 (4)	
H4A	0.6536	0.3492	0.5182	0.044*	
C5	0.67981 (13)	0.21777 (17)	0.61363 (8)	0.0333 (3)	
H5A	0.5965	0.1968	0.6227	0.040*	
C6	0.76889 (12)	0.15677 (16)	0.66330 (7)	0.0273 (3)	
C7	0.72692 (12)	0.05618 (16)	0.72799 (7)	0.0277 (3)	
C8	0.82354 (13)	−0.01705 (17)	0.78080 (8)	0.0313 (3)	
H8A	0.8882	−0.0650	0.7499	0.038*	
H8B	0.8611	0.0669	0.8110	0.038*	
F1	0.87045 (9)	0.42468 (11)	0.47751 (5)	0.0478 (3)	

### Atomic displacement parameters (Å^2^)

**Table:**

	*U*^11^	*U*^22^	*U*^33^	*U*^12^	*U*^13^	*U*^23^
O1	0.0225 (6)	0.0481 (6)	0.0370 (5)	−0.0033 (4)	−0.0003 (4)	−0.0021 (5)
N1	0.0337 (7)	0.0341 (7)	0.0405 (7)	0.0037 (5)	0.0029 (6)	0.0004 (5)
N2	0.0346 (7)	0.0329 (7)	0.0333 (6)	−0.0019 (5)	−0.0049 (6)	0.0031 (5)
N3	0.0413 (8)	0.0548 (9)	0.0392 (7)	0.0024 (7)	0.0065 (6)	0.0069 (6)
C1	0.0248 (7)	0.0338 (8)	0.0328 (7)	0.0029 (6)	−0.0019 (6)	−0.0054 (6)
C2	0.0293 (8)	0.0374 (8)	0.0376 (7)	−0.0015 (6)	0.0049 (6)	−0.0064 (6)
C3	0.0414 (9)	0.0314 (7)	0.0299 (7)	−0.0013 (6)	0.0036 (6)	−0.0031 (6)
C4	0.0355 (9)	0.0378 (8)	0.0366 (8)	0.0021 (6)	−0.0068 (6)	−0.0006 (6)
C5	0.0260 (7)	0.0343 (8)	0.0394 (8)	0.0003 (6)	−0.0025 (6)	−0.0041 (6)
C6	0.0241 (7)	0.0274 (7)	0.0304 (7)	0.0011 (6)	0.0005 (5)	−0.0075 (5)
C7	0.0232 (7)	0.0287 (7)	0.0313 (7)	0.0004 (5)	−0.0009 (5)	−0.0098 (5)
C8	0.0254 (7)	0.0336 (7)	0.0348 (7)	−0.0004 (6)	0.0003 (6)	−0.0012 (6)
F1	0.0544 (6)	0.0501 (6)	0.0389 (5)	−0.0045 (4)	0.0040 (4)	0.0089 (4)

### Geometric parameters (Å, º)

**Table:**

O1—C7	1.2154 (16)	C3—C4	1.373 (2)
N1—N2	1.2425 (17)	C4—C5	1.377 (2)
N1—C8	1.4652 (19)	C4—H4A	0.9300
N2—N3	1.1296 (17)	C5—C6	1.3936 (19)
C1—C2	1.387 (2)	C5—H5A	0.9300
C1—C6	1.3942 (19)	C6—C7	1.4826 (19)
C1—H1B	0.9300	C7—C8	1.5229 (19)
C2—C3	1.370 (2)	C8—H8A	0.9700
C2—H2A	0.9300	C8—H8B	0.9700
C3—F1	1.3591 (16)		
			
N2—N1—C8	115.85 (12)	C4—C5—H5A	119.5
N3—N2—N1	170.98 (14)	C6—C5—H5A	119.5
C2—C1—C6	120.33 (13)	C5—C6—C1	119.02 (13)
C2—C1—H1B	119.8	C5—C6—C7	118.29 (12)
C6—C1—H1B	119.8	C1—C6—C7	122.69 (12)
C3—C2—C1	118.38 (13)	O1—C7—C6	121.43 (12)
C3—C2—H2A	120.8	O1—C7—C8	119.67 (12)
C1—C2—H2A	120.8	C6—C7—C8	118.90 (11)
F1—C3—C2	118.53 (13)	N1—C8—C7	113.59 (12)
F1—C3—C4	118.33 (13)	N1—C8—H8A	108.8
C2—C3—C4	123.14 (14)	C7—C8—H8A	108.8
C3—C4—C5	118.09 (14)	N1—C8—H8B	108.8
C3—C4—H4A	121.0	C7—C8—H8B	108.8
C5—C4—H4A	121.0	H8A—C8—H8B	107.7
C4—C5—C6	121.04 (13)		
			
C6—C1—C2—C3	−0.2 (2)	C2—C1—C6—C7	178.42 (12)
C1—C2—C3—F1	−178.68 (12)	C5—C6—C7—O1	−2.87 (19)
C1—C2—C3—C4	1.1 (2)	C1—C6—C7—O1	177.86 (12)
F1—C3—C4—C5	178.78 (12)	C5—C6—C7—C8	177.46 (12)
C2—C3—C4—C5	−1.0 (2)	C1—C6—C7—C8	−1.81 (18)
C3—C4—C5—C6	−0.1 (2)	N2—N1—C8—C7	−54.22 (16)
C4—C5—C6—C1	1.0 (2)	O1—C7—C8—N1	11.84 (17)
C4—C5—C6—C7	−178.34 (12)	C6—C7—C8—N1	−168.49 (11)
C2—C1—C6—C5	−0.84 (19)		

### Hydrogen-bond geometry (Å, º)

**Table:**

*D*—H···*A*	*D*—H	H···*A*	*D*···*A*	*D*—H···*A*
C1—H1*B*···O1^i^	0.93	2.45	3.3722 (17)	174

Symmetry code: (i) *x*+1/2, *y*, −*z*+3/2.
